# George Frederick Rossiter

**Published:** 1921-12

**Authors:** 


### GEORGE FREDERICK ROSSITER, M.B. Lond., M.R.C.S. Eng.

THE passing of Dr. G. F. Rossiter, of Cairo Lodge, Weston-super-Mare, ends a life that one can truly say has been well spent. He received his medical education at St. Thomas's Hospital, where he won the Cheselden and Treasurer's Gold Medal, and graduated in honours at the University of London. Coming to Weston-super-Mare in 1877, he was appointed Hon. Surgeon to the Hospital in 1880, and remained on the active staff until 1905, when he resigned, and was appointed Consulting Surgeon.

Dr. Rossiter was at one time almost as well known to the medical profession of our city as he was at Weston-super-Mare, and his work and professional opinion was highly esteemed. To Dr. Alford, of Weston-super-Mare, we are indebted for the following note :—

"He was a member of the Bristol Medico-Chirurgical Society and for many years a regular attendant at the meetings, and would have been a President of the Society, but failing health made him decline the Council's nomination for his election.

"Possessing high ideals of professional honour, he acted up to them, and spent his active life in the service of others, delighting in his work for its own sake rather than for any monetary reward it might bring him.

"A genial, unassuming man, and most considerate to his colleagues and to those who were junior to him, Rossiter was particularly thoughtful. The writer can never forget his many kindnesses, and even during the last thirteen years, when he was laid by, suffering from cardiac symptoms, he was ever ready to help and advise those who had the privilege of coming into contact with him, for he was not only a very able practitioner, but counsellor and kind friend, and there are many of his patients who are grateful to him in a multitude of ways, for his judgment, whether in professional matters or otherwise, was sound and wise.

" The end came quite suddenly and peacefully in the early morning of Advent Sunday, November 27th."

To his widow and sons we tender sincerest sympathy.

